# Intestinal Permeability Biomarkers for Predicting Cardiometabolic Risk in Type 2 Diabetes Mellitus

**DOI:** 10.3390/nu18010167

**Published:** 2026-01-04

**Authors:** Nursel Dal, Saniye Bilici, Sirin Akin, Perim Fatma Turker

**Affiliations:** 1 Department of Nutrition and Dietetics, Faculty of Health Sciences, Bandirma Onyedi Eylul University, Balikesir 10250, Türkiye; nurselsahin@bandirma.edu.tr; 2 Department of Nutrition and Dietetics, Faculty of Health Sciences, Gazi University, Ankara 06560, Türkiye; sgbilici@gazi.edu.tr; 3 Interrnal Medicine Specialist, Ministry of Health Torbali State Hospital, İzmir 35860, Türkiye; drsirinakin@gmail.com; 4 Department of Nutrition and Dietetics, Faculty of Health Sciences, Acibadem Mehmet Ali Aydinlar University, Istanbul 34752, Türkiye

**Keywords:** diabetes, cardiovascular risk, trimethylamine N-oxide, zonulin, intestinal permeability, I-FABP

## Abstract

**Background:** Diabetes can increase cardiovascular risk (CVR) through hyperglycemia and intestinal damage. The purpose of this study is to evaluate several intestinal permeability biomarkers in predicting CVR in patients with type 2 diabetes mellitus (T2DM). **Methods:** This study was conducted in 2024 with a total of 70 patients with T2DM, aged 19–64 years (32.9% men, 67.1% women). Socio-demographic data and health status were collected; Framingham Risk Score (FRS), anthropometric measures, and serum parameters (glucose, HbA1c, lipids, CRP, TNF-α, IL-6, trimetilamine-N-oxide (TMAO), zonulin, intestinal fatty acid binding protein (I-FABP)) were evaluated, and visceral adiposity index (VAI) and plasma atherogenic index (PAI) were calculated. **Results:** The mean age of patients (*n* = 70) was 55.0 ± 7.55 years. According to FRS, 18.5% of individuals were determined to be at medium–high CVR; a positive correlation was found between BMI, waist–height ratio, body fat ratio, VAI value, and FRS total score (*p* < 0.05). Serum TMAO, zonulin, and I-FABP levels did not differ between low-risk and medium–high-risk patients (*p* > 0.05). Serum TMAO, zonulin, and I-FABP levels were positively correlated with TNF-α and IL-6 levels, and serum TMAO and I-FABP levels were positively correlated with triglyceride levels (*p* < 0.05). Moreover, serum zonulin and I-FABP levels were positively correlated with PAI (*p* < 0.05). **Conclusions:** Abdominal obesity and intestinal permeability may affect inflammatory processes and blood lipids in patients with T2DM. Further studies with large samples are needed to examine dietary factors related to the relationship between intestinal permeability and cardiometabolic risk.

## 1. Introduction

Type 2 diabetes mellitus (T2DM) is a chronic metabolic disease characterized by hyperglycemia due to insulin resistance or insufficient insulin production. T2DM, a rising threat to human health worldwide, accounts for approximately 90% of all diabetes cases, and cardiovascular events are the leading cause of risk of early death in people with diabetes [1]. The study using NHANES data found that 57% of adults with T2DM had at least two cardiovascular disease (CVD) risk factors, such as hypertension, hyperlipidemia, and obesity, and 21% had all three of these risk factors [2]. Otto et al. (2023) reported that most patients with T2DM have at least one cardiovascular risk (CVR) factor, and approximately half have established CVD [3].

An unhealthy diet, especially the Western diet, has been linked to a range of chronic diseases, including obesity, T2DM, CVD, and certain cancers [4]. The Western diet is characterized by a high intake of energy-dense, nutrient-poor foods such as fast foods, soft drinks, and highly processed foods, which are high in added sugars, salt, and saturated fats. This diet can disrupt the balance and diversity of harmful and beneficial bacteria in the gut microbiome, leading to dysbiosis. Dysbiosis, or an imbalance of the intestinal microbiota, can lead to increased intestinal permeability, allowing bacterial endotoxins to enter the bloodstream and trigger inflammation [5]. Changes in gut microbiota (dysbiosis) may affect the host’s sensitivity to insulin, body weight, and lipid and carbohydrate metabolism. Dysbiosis can lead to activation of proinflammatory mechanisms, metabolic toxicity, and insulin resistance [6], promoting arterial hypertension and atherosclerosis, contributing to the development of inflammation, and increasing the risk of CVDs [7].

Consumption of the Western diet has been demonstrated to cause alterations in gut microbiota composition, and this may influence gut microbial trimetilamine production and trimetilamine-N-oxide (TMAO) synthesis [8]. High plasma TMAO levels are thought to increase the risk of CVD through various metabolic pathways such as endothelial dysfunction, foam cell formation, inflammation in vascular cells, atherosclerosis, and dysbiotic microbiome [9]. In addition, an increase in intestinal permeability due to alterations in gut microbiota composition causes many diseases, including diabetes, along with inflammation. Also, impaired intestinal barrier function has been associated with poor glycemic control and diabetes complications [5,10]. Circulating lipopolysaccharide and zonulin (a marker of intestinal permeability) levels were shown to be significantly increased in patients with T2DM and were associated with inflammatory markers such as TNF-α, IL-6, and poor glycemic/lipid control. Therefore, it has been reported that serum zonulin levels can be used as a new indicator of the relationship between chronic low-grade inflammation and intestinal permeability in persons with diabetes [10]. It has been reported that high levels of intestinal fatty acid binding protein (I-FABP), which is released into the circulation when intestinal epithelial cells are damaged, affect lipid and inflammatory responses and cause atherosclerotic plaque formation in macrophages. It has also been stated that high serum I-FABP levels may indicate the presence of obesity, insulin resistance, and T2DM and may be associated with CVR [11].

Although scores such as the Framingham Risk Score (FRS) are frequently used to estimate CVR, indices such as the visceral adiposity index (VAI), which combines anthropometric and metabolic measurements, and the plasma atherogenic index (PAI), which is not a single lipid indicator, have been used in recent years [12,13,14]. On the other hand, considering the effect of the gut microbiome on the etiology and prognosis of diabetes, the role of the gut barrier, which can cause oxidative stress, systemic infection, and inflammatory response, is very important for CVD risk. However, there is lack of studies on the relatively newer intestinal permeability biomarkers such as TMAO, zonulin, and I-FABP, which may be associated with CVD risk in patients with T2DM. Studies are either solely concerned with T2DM or CVD-inflammation [9,10,11]. Therefore, this study aimed to evaluate some intestinal permeability biomarkers (TMAO, zonulin, and I-FABP) in predicting CVD risk in patients with T2DM. The research hypotheses:

- Serum TMAO, zonulin, and I-FABP levels in patients with T2DM are associated with the FRS.
- Serum TMAO, zonulin, and I-FABP levels are associated with PAI in patients with T2DM.
- Serum TMAO levels, a metabolite of gut microorganisms, are associated with blood lipids and inflammatory biomarkers in patients with T2DM.
- Serum zonulin and I-FABP levels, markers of intestinal permeability, are associated with blood lipids and inflammatory biomarkers in patients with T2DM.

## 2. Materials and Methods

### 2.1. Participants and Study Design

The minimum sample size required for the study was calculated as 68 people with 80% power, α = 0.05 significance level, and d = 0.15 effect size in the G*Power 3.1.9.7 program. The cross-sectional study was conducted between May and December 2024. It was completed with a total of 70 volunteer patients, 23 male and 47 female, aged 19–64, who were followed up in the Internal Medicine Outpatient Clinic of a district hospital in Turkey, who had a diagnosis of T2DM for at least 6 months, and whose lifestyle, exercise, and nutritional habits had not changed in the last four weeks. Patients with the criteria specified below were not included in the study because metabolic processes change or may affect dependent and independent variables:

- Receiving insulin treatment,
- Having any CVDs other than hypertension, undergone cardiac surgery, or a history of cerebrovascular disease,
- Using antilipidemic drugs,
- Having any autoimmune disease, or a psychiatric diagnosis,
- Having liver or kidney failure, or diabetic nephropathy,
- Pregnant or breastfeeding,
- Body mass index (BMI) < 18.5 kg/m^2^ and >40 kg/m^2^.

### 2.2. Data Collection

The data collection form consists of information about the patient’s socio-demographic and health status. Blood pressure, anthropometric measurements (body weight (kg), height (cm), waist circumference (cm), waist–height ratio, and neck circumference (cm) measurements), and body composition (body fat percentage (%), body muscle mass (kg), and body water percentage (%)) were measured by a portable TANITA BC 601 branded body analysis device. Systolic and diastolic blood pressure measurements were performed with an automatic blood pressure monitor, Omron (HEM-7121), calibrated at the beginning of the study. The study also calculated VAI [12] and PAI for cardiometabolic risk assessment [13]. Biochemical parameters (fasting glucose, HbA1c, total cholesterol, triglyceride, LDL-C, HDL-C, CRP, TNF-α, IL-6, TMAO, zonulin, I-FABP), and data regarding the determination of Framingham Risk Score (FRS) were collected also. The FRS was evaluated as >20% high risk, 10–20% moderate risk, and <10% low risk [14].

### 2.3. Statistical Analysis

The obtained data were evaluated with the SPSS 26.0 statistical package program. Whether quantitative variables were suitable for normal distribution was evaluated with the skewness-kurtosis coefficient. In examining the relationship between continuous and two-group variables, a *t*-test was used for normally distributed data, and the Mann–Whitney U test was used for non-normally distributed data. Differences between groups in categorical variables were checked with the chi-square test. Pearson correlation analysis was used when normality was achieved, and Spearman analysis was used when normality was not achieved to evaluate the relationship between serum parameters and other variables. Statistical significance was evaluated at the *p* < 0.05 level.

## 3. Results and Discussion

### 3.1. Socio-Demographic Profile and Health Status

T2DM is considered a lifestyle disease and is a global health problem where epidemiological profiles such as age at diagnosis and anthropometric indicators change [15]. In this study, 30.0% of the patients were between 45–54 years of age and 60.0% were between 55–64 years of age. It was determined that 55.7% of the patients had a duration of diabetes of 0–5 years; the majority (90.0%) used oral antidiabetic agents for diabetes treatment, and only 21.4% received diabetes education. While 47.8% of males had no chronic disease other than T2DM and 30.4% had hypertension, 31.9% of females had no additional chronic disease and 66.0% had hypertension. The rate of males applying dietary therapy for T2DM was determined as 21.7% and the rate of females as 12.8% (*p* > 0.05) (Table 1).

### 3.2. The Relationship Between Obesity, Visceral Adiposity, and CVR

It is known that overweight and obesity are important risk factors for CVD. Indeed, diabesity, which expresses the strong relationship between obesity and diabetes, is shown as a direct result of the obesity epidemic. Current studies classify 50–80% of patients with T2DM as overweight or obese [15,16]. In this study, most males (65.2%) were overweight, while most females (74.4%) were obese (*p* < 0.05) (Table 1). In addition, the mean VAI used as an indicator of both abdominal obesity and CVR was found to be higher in females (7.0 ± 4.22) than in males (5.2 ± 3.50) (*p* < 0.05) (Table 2). These findings support the strong effect of obesity on the development of T2DM and the increase in the risk of CVD associated with obesity. The fact that most individuals have not received diabetes education that includes the importance of body weight control in diabetes management, that they do not follow any diet for diabetes, and especially the sedentary lifestyle of females are important reasons for this situation. In addition, considering the average age of females, it is thought that abdominal obesity together with menopause and a sedentary lifestyle negatively affects diabetes management.

### 3.3. Assessment of CVR with FRS and Its Relationship with Anthropometric Measurements

An et al. (2021) [17] reported that the median time to occurrence of any complication of T2DM ranged from 3.0 to 5.2 years. In the multinational CAPTURE study conducted in 214 centers in 13 countries with adults with T2DM, the overall weighted CVD prevalence was estimated as 34.8% [18]. In a study conducted in Bangladesh, 25.6% of individuals with T2DM aged 30 years and older were reported to be at intermediate risk and 52.3% at high risk in terms of CVR according to the FRS [19]. In a study investigating CVR in 47 T2DM patients with a mean age of 50.8 ± 5.61 years in Turkey, the total FRS mean score was found to be 8.9 ± 3.06, and 66.0% of the patients were found to be at low risk and 34.0% at high risk [20]. In this study, the total FRS average of T2DM patients was found to be 13.1 ± 3.96, and 18.5% were found to be at moderate and high CVR (Table 3). The total FRS average of females (14.3 ± 3.22) was found to be significantly higher than the average score of males (10.6 ± 4.23) (*p* < 0.05). However, males were in a higher risk group than females (*p* < 0.05). It can be said that the fact that the total FRS average of females in the study was higher than males, but males were found to be at higher CVR, may be since FRS scoring depends on factors such as age, cholesterol levels, and blood pressure, and that FRS classification varies by gender.

In a cohort study, all abdominal obesity indices (VAI, Chinese visceral adiposity index, and lipid accumulation product) were found to be positively associated with the risk of cardiovascular events in individuals with T2DM [21]. High values of VAI, an index combining anthropometric and metabolic measurements, were associated with poor glycemic control, dyslipidemia, and high triglyceride-glucose index in females. It has also been reported that VAI is a good marker of glycemic control when compared with waist circumference, BMI, or triglyceride-glucose indices [22]. In this study, no significant difference was found between low-risk and moderate–high-risk patients in terms of BMI, waist circumference, waist–height ratio, neck circumference, body fat percentage, and VAI means (*p* > 0.05) (Table 3). However, there was a positive correlation between the total FRS and the age of the individuals, body muscle mass, and body water percentage, and a positive correlation between BMI, waist–height ratio, body fat percentage, and VAI and the total FRS (Table 4). These results are consistent with the literature [23] and support the altered metabolic profile of abdominal obesity, which predicts increased cardiometabolic risk [22].

### 3.4. The Relationship Between Lipid Profile, PAI, and CVR

The most important cause of CVDs is atherosclerosis, and dyslipidemia serves as a marker of developing atherosclerosis. Khil et al. (2023) associated increases in serum total cholesterol levels from pre-diagnosis to post-diagnosis with increased CVD risk in T2DM patients, while decreases in total cholesterol levels were associated with reduced CVD risk [24]. In this study, when biochemical parameters of low-risk and moderate–high-risk patients in terms of CVR were examined, no significant difference was found between fasting glucose and HbA1c, triglyceride, LDL-C, HDL-C, CRP, TNF-α, and IL-6 levels (*p* > 0.05) (Table 3). Poor glycemic control of the patients or imbalance of numbers in the groups in terms of CVR may be a reason for this result.

In recent years, PAI has been used as a potential indicator to predict the risk of coronary artery disease instead of the predictive value of traditional single lipid parameters. Studies suggest that there is a significant relationship between PAI and obesity [25,26] and that PAI may be a new lipid marker for individuals with T2DM and coronary artery disease [27]. In a study, PAI was found to be positively associated with the risk of CVD after adjustment for age, gender, BMI, physical activity, hypertension, and dyslipidemia (OR: 1.32, 95% CI, *p* < 0.05), and it was stated that it can be used to identify high-risk individuals for CVD in the general population [28]. In another study, PAI in patients with T2DM; waist circumference (r = 0.1095, *p* < 0.05), waist-hip ratio (r = 0.1926, *p* < 0.05), CVR indices (r = 0.506, *p* < 0.05) [29]. It has also been stated that in cases where atherogenic parameters such as triglyceride and HDL-C are at normal levels, PAI can be used as a diagnostic alternative and can be used to estimate CVR and monitor the effectiveness of treatment [30]. In this study, the total cholesterol level and PAI mean of patients with moderate–high CVR (238.4 ± 45.07 mg/dL and 0.7 ± 0.15, respectively) were found to be higher than the total cholesterol level and PAI mean of patients with low risk (205.3 ± 34.85 mg/dL and 0.6 ± 0.11, respectively) (*p* < 0.05) (Table 3). In addition, a positive correlation was found between the patients’ serum triglyceride, total cholesterol, LDL-C levels, and PAI values and the FRS total score (r = 0.265, r = 0.441, 0.367, and 0.385, respectively, *p* < 0.05) (Table 4). Together, these results indicate that worsening lipid profiles are strongly reflected in PAI values, reinforcing the idea that PAI may serve as a sensitive and complementary indicator for CVR stratification.

### 3.5. The Relationship Between Intestinal Permeability, Inflammatory Parameters Biomarkers, and CVR

Current studies suggest that increased intestinal permeability may trigger dysbiosis, leading to metabolites (lipopolysaccharides, TMAO) entering the bloodstream and accelerating the development of CVD via inflammation [31,32]. A meta-analysis revealed that the intestinal barrier was damaged in patients with CVD, and markers of intestinal permeability, such as lipopolysaccharide, d-lactate, zonulin, and I-FABP, were increased [33]. Yuan et al. (2021) [34] reported that serum lipopolysaccharide level was positively correlated with HbA1c in patients with T2DM, and patients with poor glycemic control (HbA1c ≥ 7.0) had higher serum lipopolysaccharide, I-FABP, and zonulin levels. Poor glycemic control as well as increased chronic complications of diabetes have been associated with impaired intestinal barrier function [34]. In this study, serum TMAO, zonulin, and I-FABP levels of patients were found to be higher in patients with low CVR than in patients with medium–high risk, although this was not statistically significant (*p* > 0.05) (Table 3). However, according to the study results, serum TMAO, zonulin, and I-FABP levels did not differ between low-risk and medium–high-risk patients. In addition, no relationship was found between serum TMAO, zonulin, and I-FABP levels of patients and the FRS total score (*p* > 0.05) (Table 4). Contrary to the literature, these results may be since low-risk patients have higher body fat percentages and HbA1c values than medium–high-risk patients, as well as the course of diabetes. It is suggested that long-term glycemic control may have an important effect on intestinal barrier function. Additionally, the small size of the study sample may also have an impact on these results. Therefore, this relationship needs to be examined in larger populations.

The level of TMAO in the blood depends on many factors, such as diet, composition and activity of the intestinal microbiota, permeability of the gut-blood barrier, activity of liver enzymes, and rate of methylamine excretion. Studies have shown an increase in the expression of proinflammatory cytokines (TNF-α, IL-6, CRP) when serum TMAO level is elevated [35,36]. Serum TMAO level has been reported to be positively correlated with zonulin and lipopolysaccharide levels, which are biomarkers of inflammatory and endothelial dysfunction [37]. However, the results of existing studies on the relationship between serum TMAO levels and lipid levels are contradictory. Some studies show that blood lipid levels are not associated with TMAO [35,38] while others show that plasma TMAO levels are positively associated with visceral adipose tissue and lipid profiles and negatively associated with insulin sensitivity [39,40]. In this study, a positive correlation was found between serum TMAO levels of patients and triglyceride (r = 0.277, *p* < 0.001), TNF-α (r = 0.727, *p* < 0.001), IL-6 (r = 0.638, *p* < 0.001), zonulin (r = 0.544, *p* < 0.001), and I-FABP (r = 0.679, *p* < 0.001) levels (Table 5).

Intestinal dysbiosis may also cause zonulin release, which may lead to the passage of luminal contents across the epithelial barrier and the release of proinflammatory cytokines leading to increased permeability [41]. Jayashree et al. (2014) reported that circulating zonulin levels are increased in patients with T2DM and correlate with poor glycemic control and elevated inflammatory markers [10]. Zonulin has been directly associated with diabetes, insulin resistance, and lipid profile. It has been reported that serum zonulin levels in persons with diabetes are positively correlated with HOMA-IR, triglyceride, total cholesterol, and LDL-C levels and negatively correlated with HDL-C levels [42]. Supporting the literature, this study also found a positive correlation between serum zonulin levels and TNF-α (r = 0.633, *p* < 0.001), IL-6 (r = 0.643, *p* < 0.001), and TMAO (r = 0.544, *p* < 0.001) levels (Table 5).

It has been reported that serum lipopolysaccharide, zonulin, and I-FABP levels are significantly increased in persons with diabetes compared to persons without diabetes and harm glycemic control [34]. While I-FABP concentrations in the circulation are low under normal conditions, when intestinal epithelial cells are damaged and inflammation develops, the amount of I-FABP released into the circulation increases [43]. Plasma I-FABP levels were significantly increased in T2DM individuals, especially those with uncontrolled glycemic and lipid profile parameters, and were associated with fasting glucose, triglyceride, and CRP levels (*p* < 0.05) [44]. Tahapary et al. (2023) found higher serum I-FABP levels in obese and T2DM individuals compared to non-T2DM individuals, emphasizing the potential role of intestinal permeability in the pathogenesis of T2DM in obese individuals [45]. In this study, a positive correlation was found between the serum I-FABP levels of the patients and triglyceride (r = 0.333, *p* < 0.001), total cholesterol (r = 0.326, *p* < 0.001), TNF-α (r = 0.608, *p* < 0.001), IL-6 (r = 0.516, *p* < 0.001) levels, and PAI (r = 0.237, *p* < 0.05). A positive correlation was also found between the serum zonulin levels and PAI (r = 0.241, *p* < 0.05), which is an indicator of lipid profile (Table 5). The fact that most of the patients in the study were obese and many of them had abdominal obesity and poor glycemic control may also be a cause of increased intestinal permeability. It should also be noted that the correlation between zonulin and blood pressure and glucose levels is an indication that zonulin may induce and/or regulate the secretion of zonulin from blood vessels even during normal physiological conditions.

Serum TMAO and I-FABP levels were found to be positively correlated with triglyceride (r = 0.277 and 0.333, respectively, *p* < 0.05) and all three parameters with TNF-α and IL-6 levels (Table 5), suggesting that intestinal permeability may affect inflammatory processes and blood lipids in patients with T2DM. In addition, the correlation of serum zonulin and I-FABP levels with PAI may be an indicator of the relationship between intestinal permeability and lipid profile. It is not yet fully understood whether the increase in intestinal permeability is the cause, the result, or both of T2DM, and further studies with larger populations are needed on this subject.

### 3.6. Limitations

To our knowledge, this is the first clinical study to evaluate CVD risk in patients with T2DM using serum levels of TMAO, zonulin, and I-FABP, which are intestinal permeability biomarkers. It is anticipated that this study will contribute to studies on the relationship between intestinal barrier function/intestinal permeability and CVD risk. However, the use of the FRS, which includes scoring of known risk factors for CVR assessment, and the small size of the study sample, are considered limiting in the study of the relationship between new biomarkers and CVD risk. In addition, the lack of dietary assessment, which is one of the important factors affecting intestinal permeability, is an important deficiency in interpreting the relationship between CVR and biomarkers.

## 4. Conclusions

One of the main goals in diabetes management is early detection and care of CVR. The results of our study suggest that individuals should take supportive measures such as an individualized diet and regular exercise programs to control their body weight and improve their nutritional habits to reduce the risk of complications related to T2DM. In this context, dietitians should actively participate in the clinical team where diabetic patients are monitored, and patients should be encouraged to participate in diabetes education.

Considering the effect of the gut microbiome on the etiology and prognosis of diabetes, the role of the gut barrier, which can cause oxidative stress, systemic infection, and inflammatory response, should not be ignored when assessing CVD risk. However, data on intestinal permeability biomarkers and CVD risk in patients with T2DM are quite scarce. Further studies on the relationship between CVD and intestinal permeability in larger populations, incorporating dietary factors, are needed.

## Figures and Tables

**Table 1:** Information on socio-demographic, health, and general characteristics of patients.

	Male (*n* = 23)		Female (*n* = 47)		Total (*n* = 70)		*p* Value
Age (year) (X̄ ± SD)	53.3 ± 9.23		55.9 ± 6.52		55.0 ± 7.55		0.229 ^a^
	N	%	N	%	N	%	
Age (year)							
35–44	5	21.7	2	4.3	7	10.0	0.062 χ^2^ = 5.569
45–54	5	21.7	16	34.0	21	30.0	
55–64	13	56.6	29	51.7	42	60.0	
Marital status							
Single	3	13.0	6	12.8	9	12.9	0.974 χ^2^ = 0.001
Married	20	87.0	41	87.2	61	87.1	
Education level							
Not literate	-	-	2	4.3	2	2.9	<0.001 * χ^2^ = 20.530
Primary school	4	17.4	34	72.3	38	54.3	
Secondary school	5	21.8	3	6.3	8	11.4	
High school	11	47.8	6	12.8	17	24.3	
University	3	13.0	2	4.3	5	7.1	
Duration of diabetes							
0–5 years	13	56.5	26	55.3	39	55.7	0.956 χ^2^ = 0.672
5–10 years	4	17.4	11	23.4	15	21.4	
10–15 years	4	17.4	7	14.9	11	15.7	
>15 years	2	8.7	3	6.4	5	7.2	
Use of oral antidiabetic drugs							
Using	20	87.0	43	91.5	63	90.0	0.553 χ^2^ = 0.353
Not using	3	13.0	4	8.5	7	10.0	
Diabetes education							
Yes	6	26.1	9	19.1	15	21.4	0.506 χ^2^ = 0.442
No	17	73.9	38	80.9	55	78.6	
Chronic disease other than T2DM							
No disease	11	47.8	15	31.9	26	37.1	0.011 * χ^2^ = 12.446
Hypertension	7	30.4	31	66.0	38	54.2	
Thyroid disease	1	4.4	1	2.1	2	2.9	
Neurological disease	2	8.7	-	-	2	2.9	
Other (Asthma, Hepatitis B)	2	8.7	-	-	2	2.9	
Dietary therapy							
Applying	5	21.7	6	12.8	11	15.7	0.485 χ^2^ = 0.939
Not applying	18	78.3	41	87.2	59	84.3	
BMI classification							
Normal	3	13.0	2	4.3	5	7.1	<0.001 * χ^2^ = 18.082
Overweight	15	65.2	10	21.3	25	35.7	
Obese class I	1	4.4	16	34.0	17	24.3	
Obese class II	4	17.4	19	40.4	23	32.9	

* *p* < 0.05, χ^2^: Chi-square test; if *n* < 5, Fisher’s exact test. ^a^ Independent Samples *t*-test.

**Table 2:** Anthropometric measurements and body compositions of the patients according to gender, and arithmetic mean, SD, median and min-max values of BMI and VAI.

	Male (*n* = 23)		Female (*n* = 47)	
	X̄ ± SD	Median (Min–Max)	X̄ ± SD	Median (Min–Max)
Body weight (kg)	87.9 ± 15.97	84.2 (69.50–128.50)	81.9 ± 13.18	82.5 (54.70–107.70)
Height (cm)	174.7 ± 7.41	175.0 (161.00–186.00)	157.1 ± 5.64	156.0 (146.00–168.00)
BMI (kg/m^2^)	28.7 ± 4.18	27.8 (24.13–37.55)	33.1 ± 4.39	33.0 (22.47–39.38)
Waist circumference (cm)	103.8 ± 10.17	102.0 (90.00–125.00)	104.7 ± 11.72	107.0 (67.00–133.00)
Waist–height ratio	0.6 ± 0.05	0.6 (0.49–0.69)	0.7 ± 0.07	0.7 (0.45–0.82)
Neck circumference (cm)	40.9 ± 3.86	41.0 (30.00–47.00)	37.6 ± 3.17	37.5 (32.00–46.00)
Body fat percentage (%)	24.4 ± 5.24	24.9 (15.90–34.20)	40.5 ± 5.71	40.3 (23.30–51.30)
Body muscle mass (kg)	62.5 ± 8.17	60.3 (50.40–86.70)	45.6 ± 5.26	44.4 (33.50–60.60)
Body water percentage (%)	54.4 ± 3.80	53.8 (47.30–59.90)	43.6 ± 3.72	43.9 (36.30–55.90)
VAI	5.2 ± 3.50	4.6 (1.57–18.27)	7.0 ± 4.22	6.0 (1.49–23.62)

BMI: Body Mass Index, VAI: Visceral Adiposity Index.

**Table 3:** Anthropometric measurements, body composition and biochemical parameters of the patients according to FRS classification.

	FRS Classification				*p* Value
	Low Risk (*n* = 57)		Moderate and High Risk (*n* = 13)		
	X̄ ± SD	Median (Min–Max)	X̄ ± SD	Median (Min–Max)	
Anthropometric measurements and body composition					
Body weight (kg)	82.5 ± 13.81	82.1 (54.70–128.50)	89.9 ± 15.55	87.1 (69.50–122.70)	0.131 ^a^
BMI (kg/m^2^)	31.8 ± 4.68	31.4 (22.47–39.38)	31.1 ± 5.25	29.1 (24.95–39.20)	0.659 ^a^
Waist circumference (cm)	103.9 ± 11.41	104.0 (67.00–133.00)	106.3 ± 10.22	106.0 (91.00–124.00)	0.470 ^a^
Waist–height ratio	0.6 ± 0.08	0.7 (0.45–0.82)	0.6 ± 0.05	0.6 (0.52–0.69)	0.222 ^a^
Neck circumference (cm)	38.3 ± 3.70	38.0 (30.00–47.00)	40.2 ± 3.57	40.0 (36.00–47.00)	0.101 ^a^
Body fat percentage (%)	36.4 ± 8.90	38.6 (15.90–51.30)	30.1 ± 10.22	27.9 (17.80–48.30)	0.059 ^a^
Body muscle mass (kg)	49.4 ± 9.59	47.9 (33.50–86.70)	59.0 ± 9.13	57.7 (46.60–76.80)	0.003 *^a^
Body water percentage (%)	46.4 ± 5.96	44.8 (36.30–59.90)	50.5 ± 6.90	51.8 (38.60–59.60)	0.061 ^a^
VAI	6.0 ± 3.40	4.9 (1.49–14.78)	7.9 ± 6.14	5.7 (2.13–23.62)	0.385 ^b^
Biochemical parameters					
Fasting glucose (mg/dL)	147.5 ± 49.62	136.3 (91.80–354.00)	151.0 ± 42.05	135.0 (113.20–269.30)	0.602 ^b^
HbA1c (%)	7.4 ± 1.43	7.0 (5.74–12.06)	7.1 ± 1.04	7.1 (5.86–9.77)	0.661 ^b^
Triglyceride (mg/dL)	152.2 ± 58.25	147.0 (52.80–294.90)	220.3 ± 147.07	161.9 (90.80–546.50)	0.122 ^b^
Total cholesterol (mg/dL)	205.3 ± 34.85	200.0 (80.50–293.10)	238.4 ± 45.07	222.90 (170.40–308.90)	0.025 *^a^
LDL-C (mg/dL)	125.2 ± 29.13	124.4 (59.22–200.12)	148.4 ± 35.33	141.0 (102.94–216.46)	0.051 ^a^
HDL-C (mg/dL)	51.4 ± 11.37	49.0 (29.30–76.00)	45.6 ± 9.32	42.2 (35.70–63.10)	0.067 ^a^
CRP (mg/dL)	0.7 ± 1.18	0.3 (0.05–8.00)	0.6 ± 0.85	0.3 (0.07–3.32)	0.762 ^b^
TNF-α (ng/L)	153.2 ± 102.04	112.3 (78.28–480.00)	105.7 ± 20.73	104.1 (67.49–151.80)	0.062 ^b^
IL-6 (ng/L)	88.7 ± 60.47	66.6 (43.82–314.70)	64.2 ± 13.03	69.1 (35.71–79.71)	0.506 ^b^
TMAO (ng/mL)	9.5 ± 6.28	7.6 (2.00–32.00)	7.0 ± 1.48	7.4 (3.30–8.72)	0.410 ^b^
Zonulin (ng/mL)	16.2 ± 7.78	13.4 (8.21–48.00)	13.3 ± 2.23	13.7 (9.17–15.87)	0.587 ^b^
I-FABP (ng/L)	576.0 ± 292.55	517.2 (102.30–1542.00)	523.6 ± 77.45	535.7 (396.80–643.20)	0.723 ^b^
PAI	0.6 ± 0.11	0.6 (0.26–0.83)	0.7 ± 0.15	0.8 (0.47–1.09)	<0.001 *^b^

* *p* < 0.05, ^a^ Independent Samples *t*-test, ^b^ Mann–Whitney U test. FRS: Framingham Risk Score, VAI: Visceral adiposity index, HbA1c: Hemoglobin A1c, LDL-C: Low-density lipoprotein cholesterol, HDL-C: High-density lipoprotein cholesterol, CRP: C-Reactive protein, TNF-α: Tumor necrosis factor-α, IL-6: Interleukin-6, TMAO: Trimethylamine N-oxide, I-FABP: Intestinal fatty acid binding protein, PAI: Plasma atherogenic index.

**Table 4:** Relationship between total FRSs and some parameters of patients according to gender.

	FRS Total Score					
	Male (*n* = 23)		Female (*n* = 47)		Total (*n* = 70)	
	r	*p*	r	*p*	r	*p*
Age	0.742 *	<0.001	0.184	0.216	0.468 *	<0.001
Anthropometric measurements and body composition						
Body weight (kg)	0.042	0.849	−0.046	0.759	−0.096	0.431
BMI (kg/m^2^)	0.160	0.467	0.057	0.704	0.268 *	0.025
Waist circumference (cm)	0.209	0.338	−0.045	0.765	0.057	0.642
Waist–height ratio	0.319	0.137	0.020	0.894	0.292 *	0.014
Neck circumference (cm)	−0.132	0.549	0.018	0.905	−0.224	0.062
Body fat percentage (%)	0.336	0.117	0.027	0.857	0.431 *	<0.001
Body muscle mass (kg)	−0.128	0.559	−0.097	0.516	−0.410 *	<0.001
Body water percentage (%)	−0.296	0.171	−0.005	0.973	−0.420 *	<0.001
VAI	0.451 *	0.031	0.150	0.316	0.314 *	0.008
Biochemical parameters						
Fasting glucose (mg/dL)	−0.239	0.272	0.253	0.086	0.051	0.677
HbA1c (%)	−0.336	0.117	0.145	0.330	0.012	0.924
Triglyceride (mg/dL)	0.380	0.074	0.216	0.144	0.265 *	0.027
Total cholesterol (mg/dL)	0.409	0.053	0.531 *	<0.001	0.441 *	<0.001
LDL-C (mg/dL)	0.449 *	0.036	0.477 *	<0.001	0.367 *	0.002
HDL-C (mg/dL)	−0.159	0.470	−0.064	0.671	0.014	0.905
CRP (mg/dL)	−0.010	0.962	0.059	0.691	0.115	0.342
TNF-α (ng/L)	−0.109	0.621	0.019	0.899	0.070	0.566
IL-6 (ng/L)	0.067	0.762	−0.018	0.905	0.055	0.653
TMAO (ng/mL)	−0.105	0.635	−0.004	0.978	0.017	0.890
Zonulin (ng/mL)	−0.074	0.738	−0.022	0.885	−0.040	0.741
I-FABP (ng/L)	−0.013	0.951	0.038	0.801	0.013	0.917
PAI	0.650 *	<0.001	0.400 *	0.005	0.385 *	<0.001
Blood pressure						
Systolic blood pressure (mmHg)	0.191	0.384	0.308 *	0.035	0.399 *	<0.001
Diastolic blood pressure (mmHg)	0.076	0.731	0.066	0.659	0.109	0.368

* *p* < 0.05, Pearson and Spearman correlation. FRS: Framingham Risk Score, VAI: Visceral adiposity index, HbA1c: Hemoglobin A1c, LDL-C: Low-density lipoprotein cholesterol, HDL-C: High-density lipoprotein cholesterol, CRP: C-Reactive protein, TNF-α: Tumor necrosis factor-α, IL-6: Interleukin-6, TMAO: Trimethylamine N-oxide, I-FABP: Intestinal fatty acid binding protein, PAI: Plasma atherogenic index.

**Table 5:** Correlation of patients’ serum TMAO, zonulin and I-FABP levels with other biochemical parameters and VAI.

	TMAO (ng/mL)		Zonulin (ng/mL)		I-FABP (ng/L)	
	r	*p*	r	*p*	r	*p*
Fasting glucose (mg/dL)	−0.204	0.091	−0.106	0.384	−0.099	0.414
HbA1c (%)	−0.073	0.549	−0.056	0.644	−0.065	0.592
Triglyceride (mg/dL)	0.277 *	0.020	0.150	0.216	0.333 *	0.005
Total cholesterol (mg/dL)	0.084	0.491	0.136	0.261	0.326 *	0.006
LDL-C (mg/dL)	−0.042	0.730	0.102	0.403	0.213	0.079
HDL-C (mg/dL)	0.014	0.908	−0.174	0.150	−0.051	0.673
CRP (mg/dL)	0.100	0.410	0.163	0.176	0.020	0.869
TNF-α (ng/L)	0.727 *	<0.001	0.633 *	<0.001	0.608 *	<0.001
IL-6 (ng/L)	0.638 *	<0.001	0.643 *	<0.001	0.516 *	<0.001
TMAO (ng/mL)	1.0	-	0.544 *	<0.001	0.679 *	<0.001
Zonulin (ng/mL)	0.544 *	<0.001	1.0	-	0.655 *	<0.001
I-FABP (ng/L)	0.679 *	<0.001	0.655 *	<0.001	1.0	-
PAI	0.029	0.812	0.241 *	0.045	0.237 *	0.048
VAI	0.209	0.083	0.155	0.199	0.228	0.058

* *p* < 0.05, Spearman correlation. TMAO: Trimethylamine N-oxide, I-FABP: Intestinal fatty acid binding protein, HbA1c: Hemoglobin A1c, LDL-C: Low-density lipoprotein cholesterol, HDL-C: High-density lipoprotein cholesterol, CRP: C-Reactive protein, TNF-α: Tumor necrosis factor-α, IL-6: Interleukin-6, PAI: Plasma atherogenic index, VAI: Visceral adiposity index.

## Data Availability

The data presented in this study are available on request from the corresponding author due to privacy restrictions.

## Abbreviations

The following abbreviations are used in this manuscript:

CVR	Cardiovascular risk
CVD	Cardiovascular disease
T2DM	Type 2 diabetes mellitus
FRS	Framingham Risk Score
TMAO	Trimetilamine-N-oxide
I-FABP	Intestinal fatty acid binding protein
VAI	Visceral adiposity index
PAI	Plasma atherogenic index
BMI	Body Mass Index
HbA1c	Hemoglobin A1c
LDL-C	Low-density lipoprotein-cholesterol
HDL-C	High-density lipoprotein-cholesterol
CRP	C-reactive protein
TNF-α	Tumor necrosis alpha
IL-6	Interleukin 6
